# Analysis of risk factors for postoperative recurrence of stage I colorectal cancer: a retrospective analysis of a large population

**DOI:** 10.3389/fsurg.2024.1388250

**Published:** 2024-04-19

**Authors:** Jiawei Wang, Zhangfa Song

**Affiliations:** ^1^ Department of Colorectal Surgery, Sir Run Run Shaw Hospital, School of Medicine, Zhejiang University, Hangzhou, China; ^2^ Key Laboratory of Biological Treatment of Zhejiang Province, Sir Run Run Shaw Hospital, School of Medicine, Zhejiang University, Hangzhou, China; ^3^ Key Laboratory of Integrated Traditional Chinese and Western Medicine Research on Anorectal Diseases of Zhejiang Province, Sir Run Run Shaw Hospital, School of Medicine, Zhejiang University, Hangzhou, China

**Keywords:** stage I colorectal cancer (stage I CRC), recurrence, survival, radiotherapy, colorectal cancer (CRC)

## Abstract

**Background:**

Colorectal cancer (CRC) is the third most common cancer worldwide. Patients diagnosed with stage I CRC typically do not require postoperative adjuvant treatment. However, postoperative recurrence is present in at least 40% of patients with CRC and often occurs in those with stage I disease. This study aimed to elucidate the current status of recurrence and clinicopathological characteristics in patients with stage I CRC.

**Methods:**

Data of indicated patients were obtained from 18 registries in Surveillance, Epidemiology, and End Results (SEER). The multivariable Fine–Gray regression model was used to identify the mortality risk of patients. Disparities in survival were analyzed using Kaplan–Meier curves. Logistic regression was employed to identify factors associated with recurrent risk overestimation.

**Results:**

Our study indicated a recurrence rate of 15.04% (1,874/12,452) in stage I CRC cases. Notably, we identified race, age, T stage, and carcinoembryonic antigen (CEA) levels as independent risk factors for tumor recurrence, substantially impacting prognosis. Furthermore, gender, race (Black), age (>65 years), elevated CEA levels, and refusal or unknown status regarding radiotherapy significantly correlated with an adverse prognosis in patients with stage I CRC.

**Conclusions:**

We identified certain key clinicopathological features of patients with stage I CRC and demonstrated the survival benefits of radiotherapy, offering a new perspective on stage I CRC follow-up and treatment recommendations.

## Introduction

Colorectal cancer (CRC) ranks as the third most prevalent cancer globally and is the second-largest contributor to cancer-related fatalities (1, 2). Despite the implementation of population-based CRC screening in the 1990s, which led to a more than 35% reduction in CRC incidence across the general population, a concerning trend has emerged: there has been an almost two-fold increase in CRC incidence among younger adults within the same time frame (3–6).

Surgery and chemotherapy are the most common therapeutic approaches for CRC. Surgery is used for stage I CRC and yields a 5-year survival rate of approximately 90% (7). Despite advancements in surgical methodologies and postoperative monitoring, the recurrence of CRC after pathologically confirmed complete resection remains a formidable challenge. Recurrence impacts a minimum of 40% of patients with CRC, often manifesting within the initial 3 years of disease emergence (8). According to the National Comprehensive Cancer Network (NCCN) guidelines, patients diagnosed with stage I CRC typically do not require postoperative adjuvant treatment (9, 10) and are usually advised to undergo follow-up examinations every 6 months postsurgery (9). However, real-world clinical observations often indicate postoperative recurrence in patients with stage I CRC (11). Additionally, patients with large tumors, previously assumed to require chemotherapy, are occasionally diagnosed with stage I CRC (12). Consequently, this study aimed to characterize the current landscape of recurrence patterns and clinicopathological characteristics among patients with stage I CRC.

## Methods

### Patients

We obtained all data from 18 registries of the Surveillance, Epidemiology, and End Results (SEER) using SEER * Stat 8.4.1 software. Ethical approval was not required since SEER is a publicly available database. Our study focused on patients who were diagnosed with stage I CRC between 2010 and 2015 according to the *International Classification of Diseases for Oncology, 3rd edition* (ICD-O-3) site codes (C18.0–C18.9, C19.9, C20.9) and ICD-O-3 behavior codes (malignant). Extracted variables included patient identification, age at diagnosis, gender, race, tumor grade, tumor size, American Joint Committee on Cancer (seventh edition) T stage, carcinoembryonic antigen (CEA) status, surgery code, treatment (chemotherapy, radiation), vital status, and survival duration in months. We excluded patients with incomplete clinical data, those diagnosed by autopsy, or those solely reported in death certificates.

We established a variable to track local recurrence by forming a patient cohort that met our inclusion criteria. This cohort comprised individuals with an initial tumor at a specific site who later developed the same tumor at an identical location (including tumors *in situ*). during subsequent follow-up. More precisely, the identification of CRC *in situ* at the original site, based on ICD site codes, constituted the definition of a local recurrence.

### Statistical analysis

We conducted comparisons among patient demographics, tumor characteristics, and treatment details using the *χ*^2^ test. To pinpoint the independent predictors of recurrence in patients with CRC, logistic regression analysis was employed. Covariates linked to overall survival (OS) were evaluated via a multivariable Cox proportional hazards model. The proportional hazards assumption was assessed using Kaplan–Meier curves and time-dependent covariates. In instances where this assumption was violated, the time-dependent Cox model was used. The findings were presented as hazard ratios (HRs) accompanied by 95% confidence intervals (CIs). Statistical analyses were carried out using SPSS Version 22.0 (IBM Corp.) and GraphPad Prism Version 8 (GraphPad Software) software.

## Results

### Clinical characteristics of the enrolled patients

**Figure 1:**
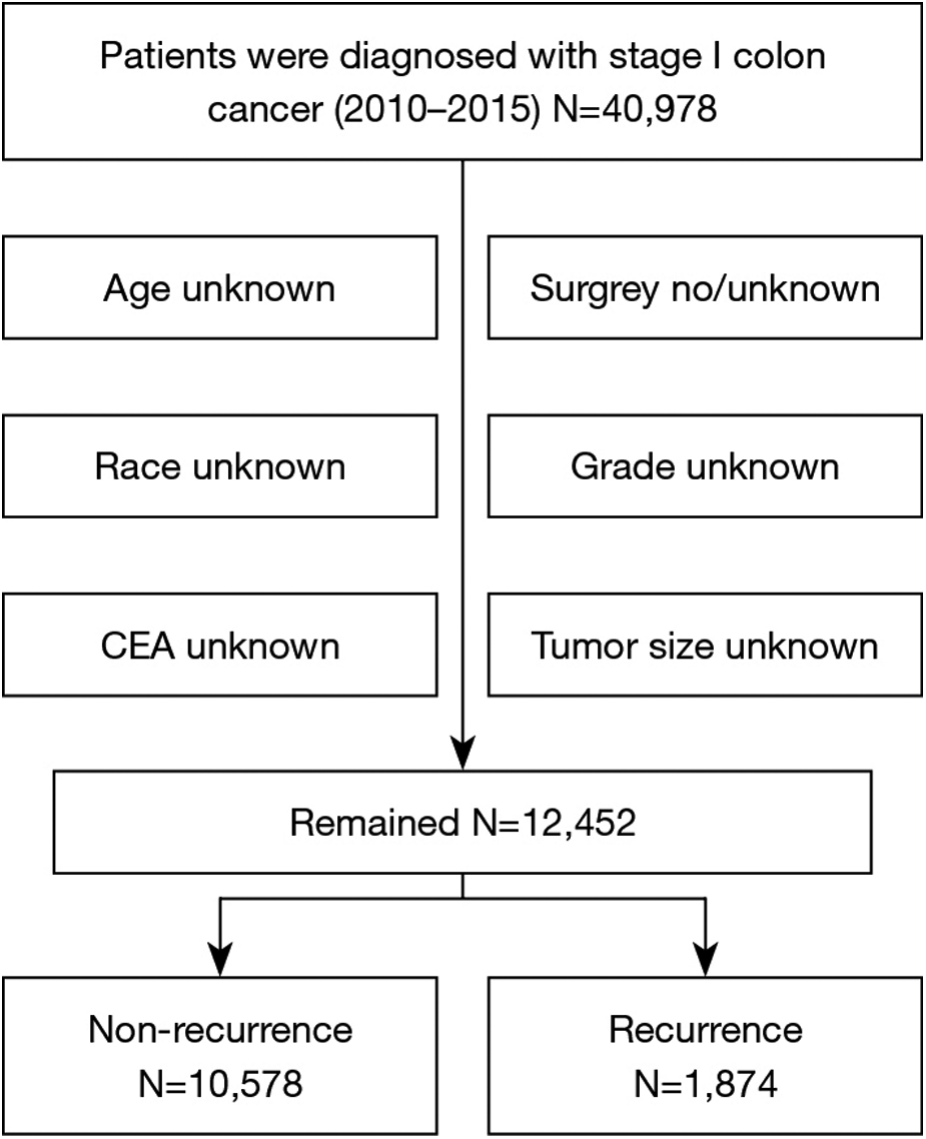
Flowchart for the surveillance, epidemiology, and end results data screening.

A total of 40,978 patients diagnosed with stage I CRC between 2010 and 2015 were included in the study, and their comprehensive clinicopathological details are presented in Table 1. All patients underwent surgical treatment. Those with incomplete information were excluded, such as in cases where race, CEA status, grade, tumor size, or surgical details were unknown (Figure 1). Upon final analysis, 12,452 patients were eligible for inclusion, comprising 10,578 patients (84.95%) with no recurrence and 1,874 patients (15.04%) with tumor recurrence.

**Table 1:** Characteristics of patients with stage I CRC.

Characteristic	Values (*N* = 40,978), *n* (%)
Gender	
Male	21,085 (51.45)
Female	19,893 (48.55)
Race	
White	31,464 (76.78)
Black	4,944 (12.07)
Other	3,995 (9.75)
Unknown	575 (1.40)
Age (years)	
<65	20,219 (49.34)
≥65	20,759 (50.66)
Tumor size (cm)	
<2	16,331 (39.85)
2–5	12,561 (30.65)
5–10	2,554 (6.23)
>10	176 (0.43)
Unknown	9,356 (22.83)
Grade	
Grade Ⅰ	8,155 (19.90)
Grade Ⅱ	24,636 (60.12)
Grade Ⅲ	2,308 (5.63)
Grade Ⅳ	370 (0.90)
Unknown	5,509 (13.44)
T stage	
T1	26,365 (64.34)
T2	14,613 (35.66)
CEA pretreatment	
Negative/normal/borderline	12,968 (31.65)
Positive/elevated	3,412 (8.33)
Unknown	24,598 (60.03)
Chemotherapy	
Yes	2,721 (6.64)
No/unknown	38,257 (93.36)
Radiotherapy	
Yes	2,524 (6.16)
Refused	95 (0.23)
Unknown	38,359 (93.61)
Radiation record	
Before surgery	1,280 (3.12)
After surgery	583 (1.42)
Before and after surgery	40 (0.10)
Recurrence	
Yes	5,935 (14.48)
No	35,043 (85.52)

Other races include Asian or Pacific Islander and American Indian/Alaska Native. CRC, colorectal cancer; CEA, carcinoembryonic antigen.

Among the 1,874 patients who experienced recurrence, 1,056 (56.35%) were male, 1,540 (82.18%) were White, 1,223 (65.26%) were aged over 65 years, and the majority were diagnosed with T2 (59.23%) disease. Additionally, 421 (22.47%) were CEA positive, 127 (6.78%) underwent chemotherapy, and 95 (5.07%) received radiotherapy. Furthermore, detailed information on tumor size and grade was collected. A comprehensive baseline analysis of clinical characteristics based on recurrence status is presented in Table 2.

**Table 2:** Characteristics of patients with stage I CRC by recurrence status.

Characteristic	Nonrecurrence (*N* = 10,578), *n* (%)	Recurrence (*N* = 1,874), *n* (%)
Gender		
Male	5,406 (51.11)	1,056 (56.35)
Female	5,172 (48.89)	818 (43.65)
Race		
White	8,397 (79.38)	1,540 (82.18)
Black	1,127 (10.65)	180 (9.61)
Other	1,054 (9.96)	154 (8.22)
Age (years)		
<65	4,767 (45.07)	651 (34.74)
≥65	5,811 (54.93)	1,223 (65.26)
Tumor size (cm)		
<2	4,057 (38.35)	745 (39.76)
2≤–<5	5,392 (50.97)	923 (49.25)
5≤–<10	1,066 (10.08)	196 (10.46)
≥10	63 (0.60)	10 (0.53)
Grade		
Grade Ⅰ	1,404 (13.27)	235 (12.54)
Grade Ⅱ	8,296 (78.43)	1,476 (78.76)
Grade Ⅲ	748 (7.07)	142 (7.58)
Grade Ⅳ	130 (1.23)	21 (1.12)
T stage		
T1	4,328 (40.92)	764 (40.77)
T2	6,250 (59.08)	1,110 (59.23)
CEA pretreatment		
Negative/normal/borderline	8,556 (80.88)	1,453 (77.53)
Positive/elevated	2,022 (19.12)	421 (22.47)
Chemotherapy		
Yes	724 (6.84)	127 (6.78)
No/unknown	9,854 (93.16)	1,747 (93.22)
Radiotherapy		
Yes	665 (6.29)	95 (5.07)
Refused	19 (0.18)	7 (0.37)
Unknown	9,894 (93.53)	1,772 (94.56)
Radiation record		
Before surgery	485 (4.58)	56 (2.99)
After surgery	160 (1.51)	36 (1.92)
Before and after surgery	15 (0.14)	3 (0.16)

Other races include Asian or Pacific Islander and American Indian/Alaska Native. CRC, colorectal cancer; CEA, carcinoembryonic antigen.

### Mortality risk analysis of stage I CRC

**Figure 2:**
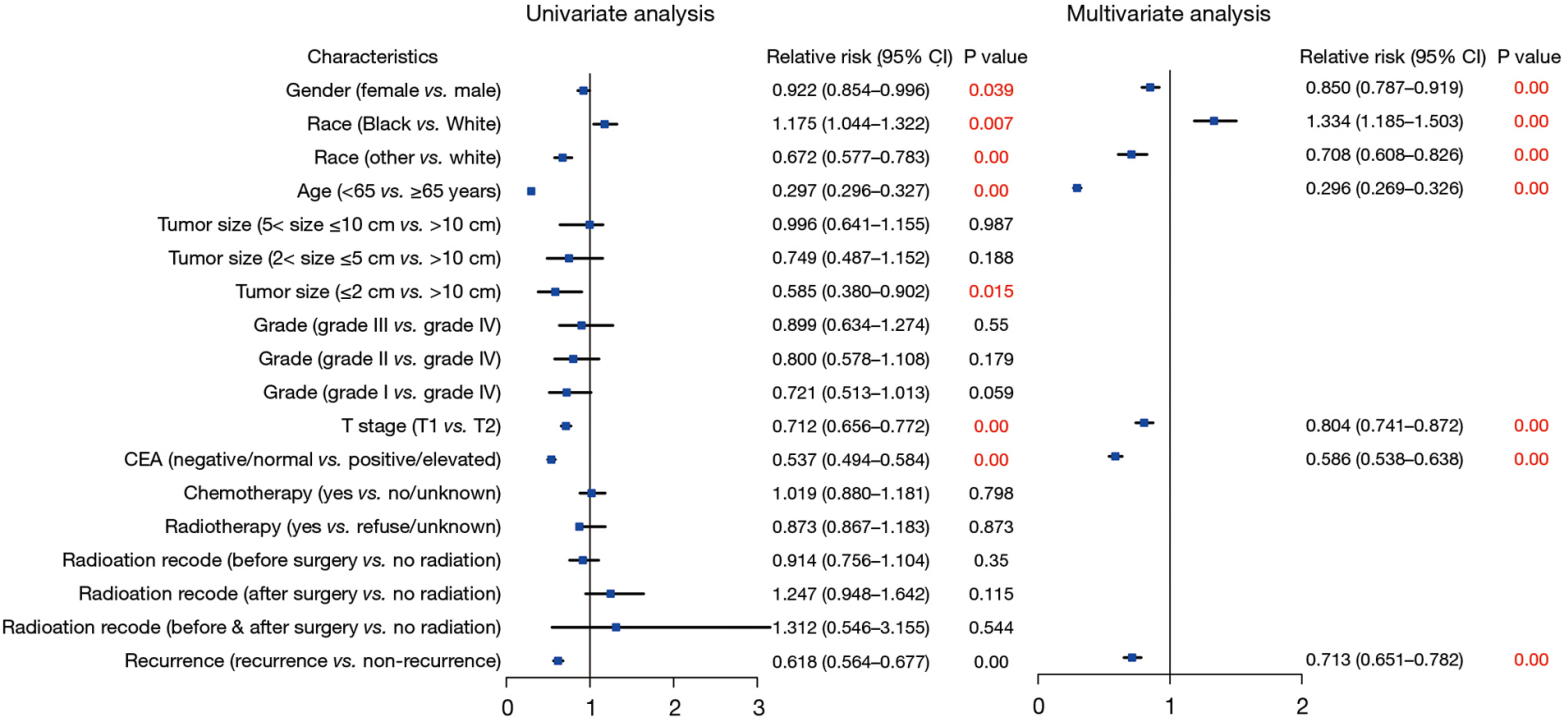
Forest plot of Cox regression analysis for assessing the risks associated with survival in patients with stage I CRC. CRC, colorectal cancer.

To investigate the factors influencing survival in stage I CRC, we conducted both univariate and multivariate Cox regression analyses. Figure 2 illustrates the associations with an overestimation of mortality risk. Male gender exhibited a higher risk of mortality overestimation in comparison to female gender (female vs. male: HR = 0.850; 95% CI: 0.787–0.919; *P* < 0.01). Moreover, Black individuals demonstrated a greater likelihood of mortality risk overestimation (Black vs. White: HR = 1.334; 95% CI: 1.185–1.503; *P* < 0.01), and other races were associated with a significant protective effect (other vs. White: HR = 0.708; 95% CI: 0.608–0.826; *P* < 0.01). Age over 65 years emerged as an independent risk factor (<65 vs. ≥65 years: HR = 0.296; 95% CI: 0.269–0.326; *P* < 0.01). Additionally, patients classified as T2 exhibited a heightened mortality risk compared to those classified as T1 (T1 vs. T2: HR = 0.804; 95% CI: 0.741–0.872; *P* < 0.01). Elevated pretreatment CEA levels were also linked to increased mortality risk in contrast to normal CEA levels (negative/normal vs. positive/elevated: HR = 0.586; 95% CI: 0.538–0.638; *P* < 0.01). Furthermore, individuals who experienced recurrence demonstrated a higher mortality risk compared to those without recurrence (nonrecurrence vs. recurrence: HR = 0.713; 95% CI: 0.651–0.782; *P* < 0.01).

**Figure 3:**
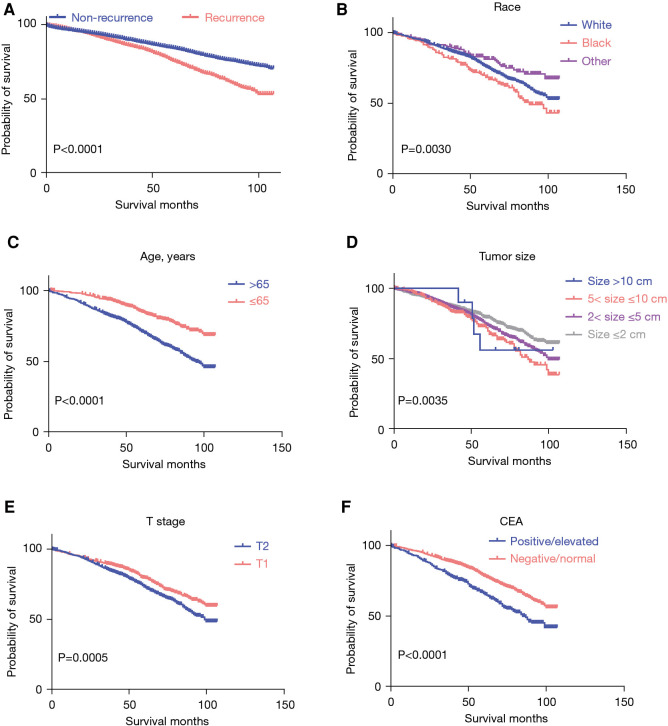
The Kaplan–Meier survival curve of patients with stage I CRC. (**A**) The Kaplan–Meier survival curve by recurrence status; (**B-F**) the Kaplan–Meier survival curve by race, age, tumor size, T stage, and CEA levels. CRC, colorectal cancer; CEA, carcinoembryonic antigen.

Additional analysis with the Kaplan–Meier survival curve highlighted significantly worse survival rates among patients with stage I CRC with recurrent lesions (Figure 3A). In patients with recurrent CRC, those of Black race (*P* = 0.0030), age over 65 years (*P* < 0.0001), with a tumor size of >5 and ≤10 cm (*P* = 0.0035), T2 stage (*P* = 0.0005), and positive/elevated CEA levels (*P* < 0.0001) exhibited a significantly poorer survival (Figure 3). The Kaplan–Meier survival curve analysis, conducted based on gender (*P* = 0.1932), grade (*P* = 0.8925), chemotherapy (*P* = 0.4963), radiotherapy (*P* = 0.5098), and radiation recode (*P* = 0.8758), revealed no significant differences (Supplementary Figure S1).

### Recurrence risk analysis of patients with stage I CRC

**Figure 4:**
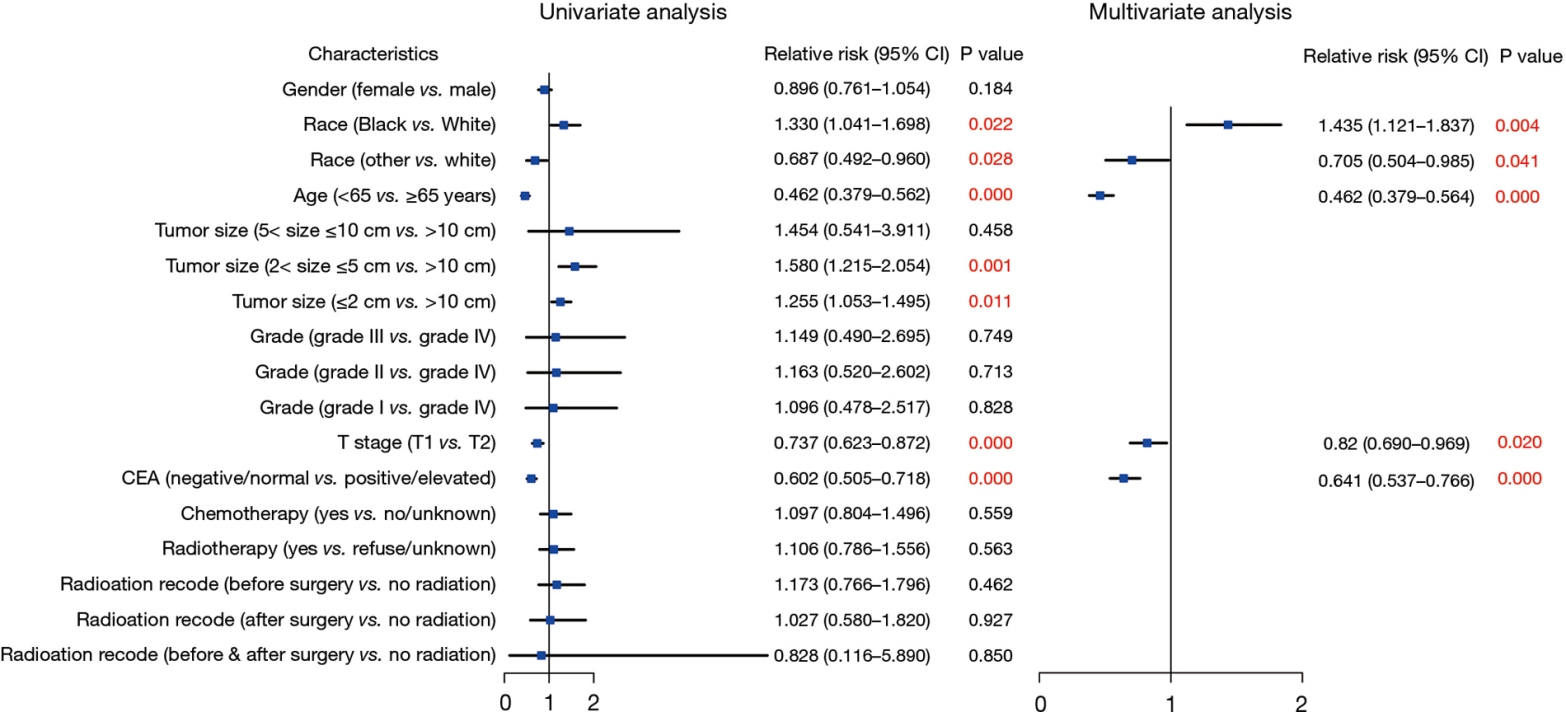
Forest plot of Cox regression analysis to assess the risks associated with survival in patients with recurrence.

A subsequent Cox regression analysis was conducted to assess the risks associated with survival in patients with CRC recurrence. The findings revealed that several factors were independently associated with the survival of patients with recurrent stage I CRC: age (<65 vs. ≥65 years: HR = 0.462; 95% CI: 0.3379–0.564; *P* < 0.001), race (Black vs. White: HR = 1.435; 95% CI: 1.121–1.837, *P* = 0.004; other vs. White: HR = 0.705; 95% CI: 0.504–0.985, *P* = 0.041), T stage (T1 vs. T2: HR = 0.82; 95% CI: 0.690–0.969, *P* = 0.020), and CEA status (negative/normal vs. positive/elevated: HR = 0.641; 95% CI: 0.537–0.766; *P* < 0.0001) (Figure 4).

**Figure 5:**
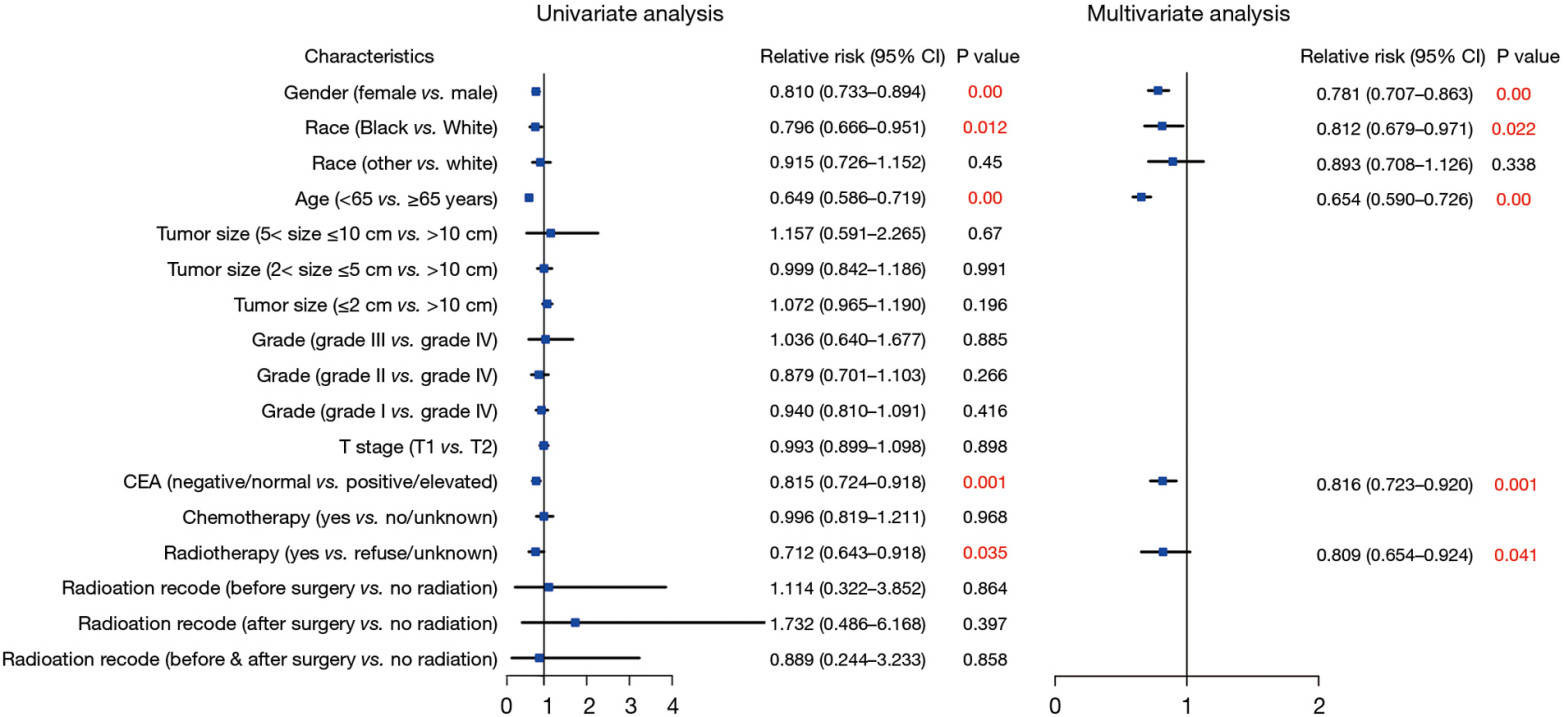
Forest plot of univariate and multivariate logistic regression analysis to assess the risks associated With recurrence.

Next, to explore risks associated with tumor recurrence among patients with stage I CRC, we conducted univariate and multivariate logistic regression analyses. Our findings indicated that female gender (HR = 0.781; 95% CI: 0.707–0.863; *P* < 0.01), Black race (HR = 0.812; 95% CI: 0.679–0.971; *P* = 0.022), age under 65 years (HR = 0.654; 95% CI: 0.590–0.726; *P* < 0.01), a negative/normal CEA level (HR = 0.816; 95% CI: 0.723–0.920; *P* = 0.001), and radiotherapy (HR = 0.809; 95% CI: 0.654–0.924; *P* = 0.041) were associated with a decreased likelihood of recurrence among patients with stage I CRC (Figure 5).

## Discussion

Cancer recurrence stands as a primary concern during the follow-up phase for patients diagnosed with CRC. More than 50% of patients with CRC experience recurrence within 2 years after surgery (13, 14), which is considered one of the main factors affecting patient prognosis. Preventing tumor recurrence can significantly improve the prognosis of these patients. While the consensus holds that patients with stage I CRC typically do not require postoperative adjuvant treatment but should be followed up every 6 months postsurgery, clinical observations often reveal postoperative recurrences among these patients. Hence, there is a need to analyze the current landscape of recurrence patterns and clinicopathological characteristics among patients with stage I CRC to inform appropriate follow-up and therapeutic strategies for early recurrence detection and curative treatment.

In previous research, the recurrence rate of CRC was reported as infrequent, ranging between 2.4% and 10% (15–19). In our study group, we observed a recurrence rate of 15% in stage I CRC, notably surpassing the rates reported in earlier research. Our results suggest that the recurrence rate of stage I CRC has increased, which may mean a larger burden on the treatment of CRC. Moreover, our study notably identified that Black and other non-White races, age over 65 years, and T2 stage were independent risk factors for tumor recurrence. These findings underscore the necessity for heightened vigilance during follow-up to mitigate recurrence or to promptly detect its onset in patients with these characteristics. Despite Nicholson et al.'s research indicating that CEA alone lacks the requisite sensitivity, even with a low threshold for recurrence detection in CRC patients (20), elevated CEA levels emerged as an independent risk factor for stage I CRC recurrence in our study. Interestingly, we identified a tumor size ≤5 cm as an independent risk factor for tumor recurrence in stage I CRC, which is somewhat counterintuitive. According to the NCCN Clinical Practice Guidelines in Oncology, patients with stage I CRC usually do not need to receive radiotherapy (9, 10). Another noteworthy finding of our study was that although radiotherapy could not reduce tumor recurrence in patients, it was significantly associated with an improved prognosis. This discovery offers a new perspective on whether radiotherapy should be recommended for patients with early-stage CRC.

Radiotherapy alone may be considered a curative approach in select cases of early-stage rectal cancer, particularly for T1N0M0 tumors, where it can achieve significant local control with minimal adverse effects. This treatment strategy allows for the preservation of the rectum and avoids the morbidity associated with surgery. Furthermore, we acknowledge the limitations of this approach, including the potential for local recurrence and the need for vigilant follow-up and management strategies to monitor and address any adverse effects.

In conclusion, we identified certain specific clinicopathological features for patients with stage I CRC and demonstrated the survival benefits of radiotherapy. These findings provide us with a new perspective on stage I CRC follow-up and treatment recommendations.

## Data Availability

The original contributions presented in the study are included in the article/**Supplementary Material**, further inquiries can be directed to the corresponding author.

## Supplementary material

Supplementary Figure S1 The Kaplan–Meier survival curve of patients with stage I CRC by gender, grade, chemotherapy, radiotherapy, and radiation recode. CRC, colorectal cancer.

## References

[1] Siegel RL Miller KD Wagle NS Jemal A. Cancer statistics, 2023. CA Cancer J Clin. (2023) 73:17–48. 10.3322/caac.21763 36633525

[2] Shin AE Giancotti FG Rustgi AK. Metastatic colorectal cancer: mechanisms and emerging therapeutics. Trends Pharmacol Sci. (2023) 44:222–36. 10.1016/j.tips.2023.01.003 36828759 PMC10365888

[3] Stoffel EM Murphy CC. Epidemiology and mechanisms of the increasing incidence of colon and rectal cancers in young adults. Gastroenterology. (2020) 158:341–53. 10.1053/j.gastro.2019.07.055 31394082 PMC6957715

[4] Bailey CE Hu CY You YN Bednarski BK Rodriguez-Bigas MA Skibber JM Increasing disparities in the age-related incidences of colon and rectal cancers in the United States, 1975–2010. JAMA Surg. (2015) 150:17–22. 10.1001/jamasurg.2014.1756 25372703 PMC4666003

[5] Murphy CC Sandler RS Sanoff HK Yang YC Lund JL Baron JA. Decrease in incidence of colorectal cancer among individuals 50 years or older after recommendations for population-based screening. Clin Gastroenterol Hepatol. (2017) 15:903–9.e906. 10.1016/j.cgh.2016.08.037 27609707 PMC5337450

[6] Siegel RL Fedewa SA Anderson WF Miller KD Ma J Rosenberg PS Colorectal cancer incidence patterns in the United States, 1974–2013. J Natl Cancer Inst. (2017) 109(8):djw322. 10.1093/jnci/djw322 28376186 PMC6059239

[7] Siegel RL Miller KD Fuchs HE Jemal A. Cancer statistics, 2021. CA Cancer J Clin. (2021) 71:7–33. 10.3322/caac.21654 33433946

[8] Renouf DJ Woods R Speers C Hay J Phang PT Fitzgerald C Improvements in 5-year outcomes of stage II/III rectal cancer relative to colon cancer. Am J Clin Oncol. (2013) 36:558–64. 10.1097/COC.0b013e318256f5dc 22868238

[9] Benson AB Venook AP Al-Hawary MM Arain MA Chen YJ Ciombor KK Colon cancer, version 2.2021, NCCN clinical practice guidelines in oncology. J Natl Compr Cancer Netw. (2021) 19:329–59. 10.6004/jnccn.2021.0012 33724754

[10] Benson AB Venook AP Al-Hawary MM Azad N Chen YJ Ciombor KK Rectal cancer, version 2.2022, NCCN clinical practice guidelines in oncology. J Natl Compr Cancer Netw. (2022) 20:1139–67. 10.6004/jnccn.2022.0051 36240850

[11] Wu Q Chen P Shu C Chen L Jin Z Huang J Survival outcomes of stage I colorectal cancer: development and validation of the ACEPLY model using two prospective cohorts. BMC Med. (2023) 21:3. 10.1186/s12916-022-02693-7 36600277 PMC9814451

[12] Chakrabarti S Peterson CY Sriram D Mahipal A. Early stage colon cancer: current treatment standards, evolving paradigms, and future directions. World J Gastrointest Oncol. (2020) 12:808–32. 10.4251/wjgo.v12.i8.808 32879661 PMC7443846

[13] Obrand DI Gordon PH. Incidence and patterns of recurrence following curative resection for colorectal carcinoma. Dis Colon Rectum. (1997) 40:15–24. 10.1007/BF02055676 9102255

[14] Sargent D Sobrero A Grothey A O'Connell MJ Buyse M Andre T Evidence for cure by adjuvant therapy in colon cancer: observations based on individual patient data from 20,898 patients on 18 randomized trials. J Clin Oncol. (2009) 27:872–7. 10.1200/JCO.2008.19.5362 19124803 PMC2738431

[15] Gilardoni E Bernasconi DP Poli S Garancini M Luperto M Zucchini N Surveillance for early stages of colon cancer: potentials for optimizing follow-up protocols. World J Surg Oncol. (2015) 13:260. 10.1186/s12957-015-0674-7 26311420 PMC4551712

[16] Kang SI Kim DW Kwak Y Lee HS Kim MH Kim MJ The prognostic implications of primary tumor location on recurrence in early-stage colorectal cancer with no associated risk factors. Int J Colorectal Dis. (2018) 33:719–26. 10.1007/s00384-018-3031-9 29594445

[17] Kim C Kim WR Kim KY Chon HJ Beom SH Kim H Predictive nomogram for recurrence of stage I colorectal cancer after curative resection. Clin Colorectal Cancer. (2018) 17:e513–8. 10.1016/j.clcc.2018.03.011 29661621

[18] Leijssen LGJ Dinaux AM Kinutake H Bordeianou LG Berger DL. Do stage I colorectal cancers with lymphatic invasion require a different postoperative approach? J Gastrointest Surg. (2019) 23:1884–92. 10.1007/s11605-018-4054-9 30511134

[19] Teloken PE Ransom D Faragher I Jones I Gibbs P Platell C. Recurrence in patients with stage I colorectal cancer. ANZ J Surg. (2016) 86:49–53. 10.1111/ans.13254 26235683

[20] Nicholson BD Shinkins B Pathiraja I Roberts NW James TJ Mallett S Blood CEA levels for detecting recurrent colorectal cancer. Cochrane Database Syst Rev. (2015) 2015:Cd011134. 10.1002/14651858.CD011134.pub2 26661580 PMC7092609

