# Supplementary figures and images for: Analysis of risk factors for postoperative recurrence of stage I colorectal cancer: a retrospective analysis of a large population
Source: Front Surg. 2024 Apr 19;11:1388250. doi: 10.3389/fsurg.2024.1388250 (PMC11072714; doi:10.3389/fsurg.2024.1388250)

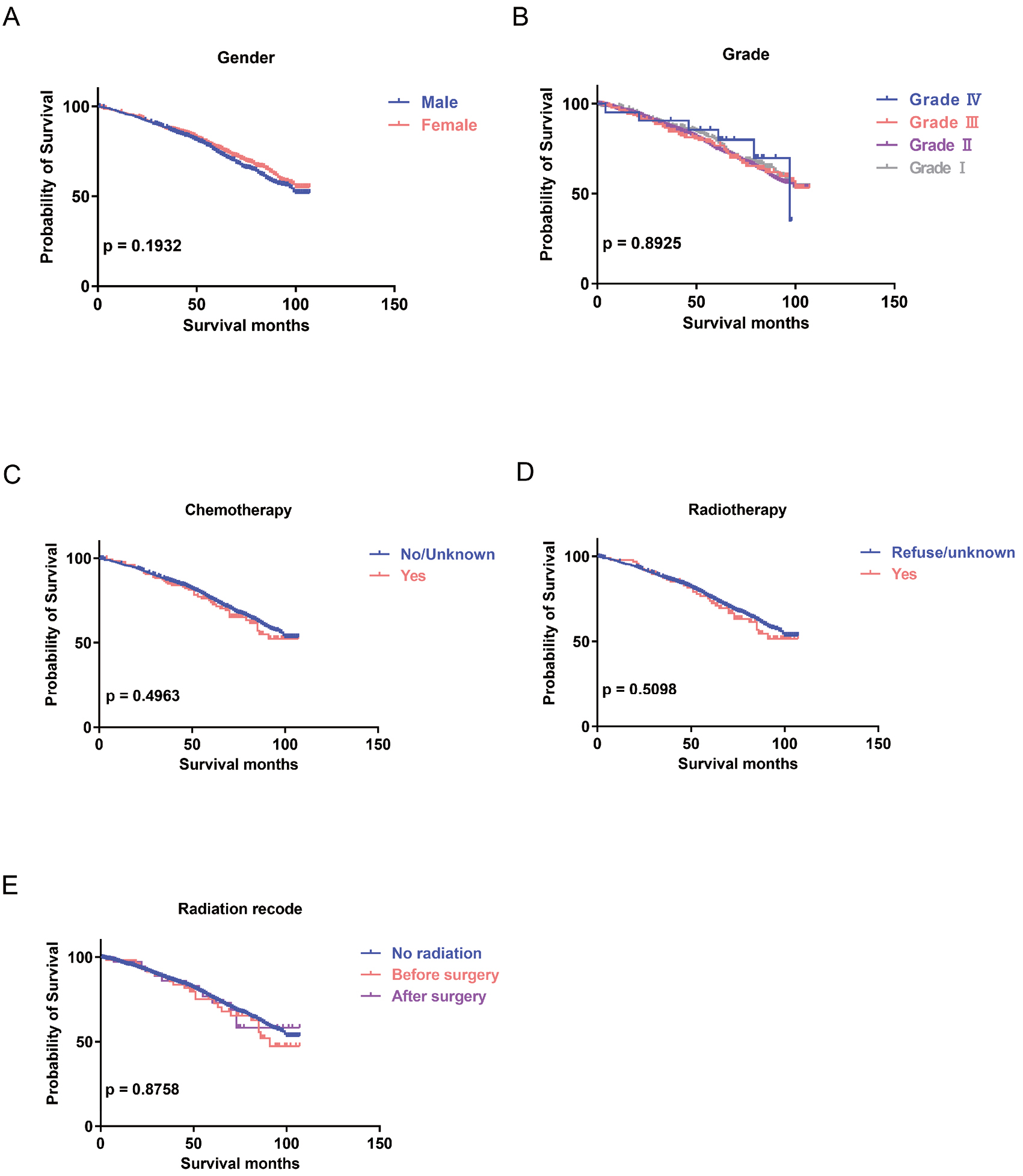

Supplement: Supplementary Figure S1 — The Kaplan–Meier survival curve of patients with stage I CRC by gender, grade, chemotherapy, radiotherapy, and radiation recode. CRC, colorectal cancer. [file Image1.jpg]
